# Osteochondral Autograft Transplantation for Subchondral Insufficiency Fracture of the Metatarsal Head in Middle-Aged and Elderly Patients: A Report of Three Cases

**DOI:** 10.7759/cureus.81390

**Published:** 2025-03-28

**Authors:** Kanu Shimokawa, Hidenori Matsubara, Junsuke Nakase, Toshifumi Hikichi, Satoru Demura

**Affiliations:** 1 Orthopaedic Surgery, Kanazawa University Hospital, Kanazawa, JPN

**Keywords:** elderly female, freiberg's disease, metatarsal bone fracture, osteochondral autograft transplantation, subchondral insufficiency fracture

## Abstract

Freiberg disease, characterized by osteochondrosis affecting the metatarsal head, typically afflicts adolescent athletes. However, subchondral insufficiency fractures of the metatarsal head, presenting similarly to Freiberg disease, occur in middle-aged and elderly individuals and pose unique challenges in diagnosis and treatment. This study reports three cases of osteochondral autograft transplantation (OAT) for collapsed metatarsal heads resembling Freiberg disease in middle-aged and elderly women with successful outcomes.

Case 1 involved a 59-year-old woman with severe metatarsophalangeal joint (MTPJ) pain and restricted range of motion (ROM) in the second toe. Case 2 featured a 66-year-old woman with trauma-induced deformity and cystic changes in the second metatarsal head. Case 3 included a 71-year-old woman with a crushed third metatarsal head and osteophyte formation. Hallux valgus was observed in one patient; the other two had a history of minor trauma. Radiological assessments revealed deformity, with MRI indicating bone marrow edema in the metatarsal head. Histological findings showed no necrosis, differentiating these cases from classical Freiberg disease. Based on these findings, OAT was chosen to restore joint function and minimize complications such as metatarsal shortening. In all cases, metatarsal head reconstruction was performed using an osteochondral column harvested from the femoral condyle. All patients experienced significant pain relief, improved ROM, and favorable imaging findings. No pain recurrence was noted during the three to seven years' follow-up period after surgery.

While classical Freiberg disease involves aseptic necrosis often attributed to repetitive stress, insufficiency fractures may arise from shear stress or subtle injury. Our cases demonstrated the effectiveness of OAT in managing advanced subchondral insufficiency fractures, even in elderly patients, by minimizing risks such as metatarsal shortening and compromised joint mobility.

## Introduction

Freiberg disease, characterized by osteochondrosis of the metatarsal head, is believed to result from aseptic necrosis induced by repetitive mechanical stress, though, its etiology remains unclear [1]. The disease often occurs in teenage girls, and much less frequently in boys, during skeletal growth [2,3]. Although cases of the disease in middle-aged and elderly patients have also been reported, recent suggestions propose treating adult-onset cases as a separate disease from classical Freiberg disease [4-6]. In Freiberg disease, Smillie's classification, which divides the progression of the disease into five stages, is the most common, and treatment is carried out according to the stage of the disease [7]. However, the best surgical procedure for this disease is still a matter of debate, and there is even less consensus on treatment for cases of middle-aged and elderly women. In our study, we performed osteochondral autograft transplantations (OATs) in one middle-aged and two elderly women presenting with metatarsal head lesions resembling Freiberg disease.

## Case presentation

Case 1

**Figure 1:**
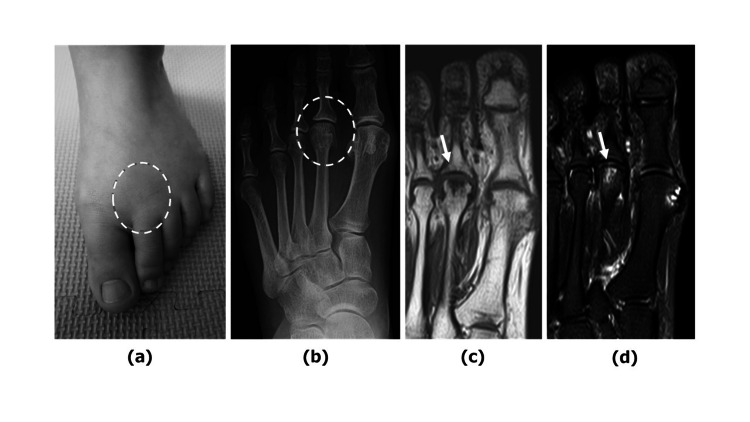
Case 1—Preoperative examination (a) Clinical photograph taken during the initial examination (dotted circle highlights the area of tenderness) (b) AP view radiograph demonstrating flattening deformity of the second metatarsal head (indicated by a dotted circle) (c) MRI T1-weighted image showing low signal in the second metatarsal head (indicated by an arrow) (d) MRI fat-suppressed T2-weighted image showing high signal in the second metatarsal head (indicated by an arrow) AP: Anteroposterior

**Figure 2:**
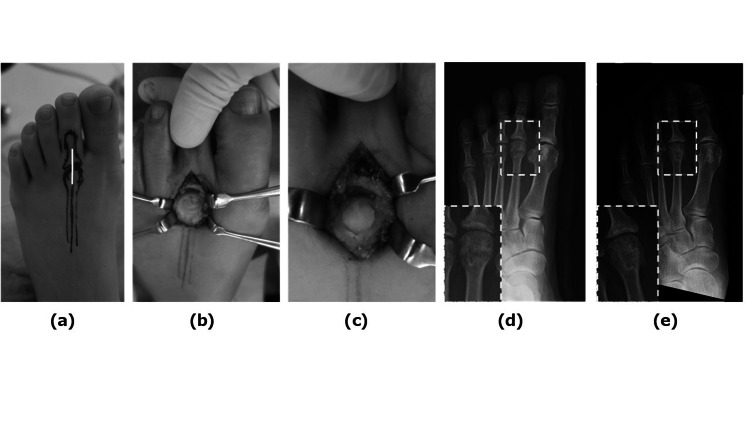
Case 1—Intraoperative photographs (a-c) and postoperative radiographs (d-e) (a) A longitudinal skin incision was made directly over the MTPJ (the incision line is indicated by a white line) (b) Severe deficiency observed in the cartilaginous surface (c) Results following OAT (d) AP view of the foot immediately postoperatively (the area enclosed by a dotted square is enlarged and shown in the lower left) (e) Radiograph one year postoperatively: Healthy osteochondral column with unchanged joint space size (the area enclosed by a dotted square is enlarged and shown in the lower left) AP: Anteroposterior; MTPJ: Metatarsophalangeal joint; OAT: Osteochondral autograft transplantation

Our first case involved a 59-year-old woman presenting to our department with a 10-month history of pain in the metatarsophalangeal joint (MTPJ) of the left second toe. Her history included prior surgery on the right hallux, and she had mild deformity with a left hallux valgus angle measuring 23°. Upon clinical examination of the left second MTPJ, tenderness was observed on the plantar aspect, and her range of motion (ROM) was limited to 30° dorsiflexion and 0° plantar flexion (Figure 1a). The patient reported a visual analog scale (VAS) score of 8.0 points. Radiographic evaluation revealed deformity of the second metatarsal head (Figure 1b), while MRI findings showed low signal on T1-weighted images and high signal on T2-weighted images in the metatarsal head (Figures 1c, 1d). These imaging findings were consistent with Smillie's stage 3 Freiberg disease. The patient underwent OAT for collapse of the left second metatarsal head, whereby a longitudinal section of the joint capsule was made from the dorsal side to conform to the articular surface of the MTPJ (Figure 2a). Due to severe deficiency of the cartilaginous surface of the metatarsal head (Figure 2b), an osteochondral column (8 mm diameter, 10 mm length) was harvested from the non-weight-bearing site of the left medial femoral condyle and grafted by drilling to match the size of the metatarsal head (Figure 2c). Histological diagnosis of the resected bone fragments showed mild bone thickening but no necrosis. The patient initiated weight-bearing with a brace at 4 weeks postoperatively; by 2 months, her VAS score improved to 2.0 points, and by 6 months, her MTPJ ROM improved to 45° dorsiflexion and 5° plantar flexion. Radiographs taken one year postoperatively revealed complete attachment of the osteochondral column, with no change in joint space size (Figures 2d, 2e). The Japanese Society for Surgery of the Foot (JSSF) lesser toe scale improved from 64 points preoperatively to 95 points at one year postoperatively [8]. At four years postoperatively, the patient remained pain-free.

Case 2

**Figure 3:**
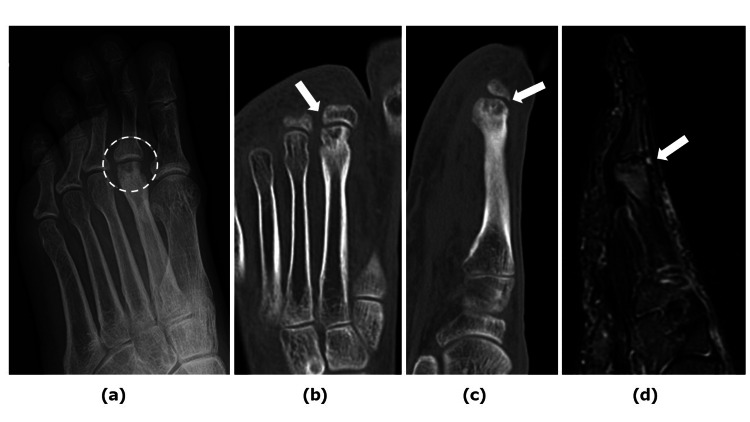
Case 2—Preoperative examination (a) Medial oblique view radiograph showing deformity and cysts of the second metatarsal head (indicated by a dotted circle) (b) Axial plane CT showing deformity and cysts of the second metatarsal head (an arrow indicates cyst) (c) Sagittal plane CT scan revealing deformity and cysts of the second metatarsal head (an arrow indicates cyst) (d) MRI T2-weighted image demonstrating high signal intensity in the third metatarsal head (an arrow indicates cyst)

**Figure 4:**
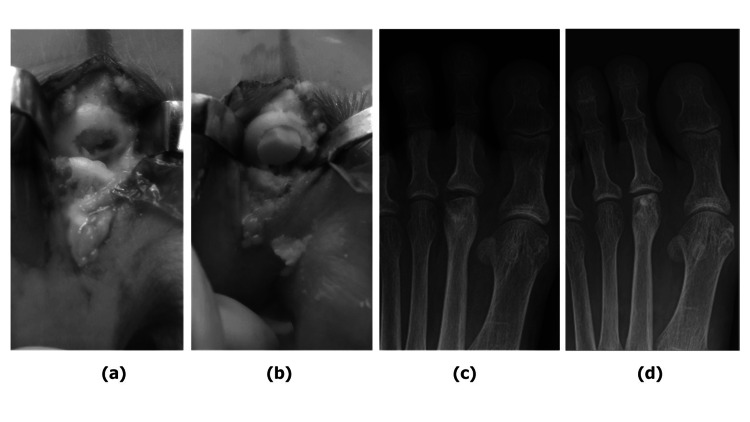
Case 2—Intraoperative photographs and postoperative radiograph (a) Many osteophytes and joint mice are found around the metatarsal bone head (b) Results following OAT (c) Immediate AP view of the foot immediately postoperatively (d) AP view of the foot three years postoperatively AP: Anteroposterior; OAT: Osteochondral autograft transplantation

Our second case involved a 66-year-old woman who had sustained a fracture to her left fourth toe one year prior to her initial visit, which was managed conservatively. Subsequently, she experienced pain in the MTPJ of the left second toe, leading to referral to our department. Physical examination revealed localized swelling and tenderness at the left MTPJ of the second toe, accompanied by restricted ROM of 15° dorsiflexion and 5° plantar flexion. Radiographic and CT imaging revealed deformity and cysts on the second metatarsal head (Figures 3a-3c), while MRI showed bone marrow edema in the metatarsal head (Figure 3d). These findings were consistent with Smillie's stage 3 Freiberg disease. Additionally, the patient’s lumbar spine and femur bone mineral density were measured at 58% and 72%, respectively, relative to the Young Adult Mean (YAM) value. The patient underwent OAT for collapse of the left second metatarsal head, revealing thickened joint synovium and detached cartilage from the joint surface (Figure 4a). Following synovectomy and curettage, an osteochondral column (6.5 mm diameter, 10 mm length) was harvested from a non-weight-bearing site of the left medial femoral condyle and implanted onto the metatarsal head (Figures 4b, 4c). The patient could bear weight on her heel with a brace one week postoperatively, progressing to full weight-bearing by six weeks. At one year postoperatively, the MTPJ ROM improved to 45° dorsiflexion and 10° plantar flexion. The JSSF scale improved from 64 points preoperatively to 95 points one year postoperatively. At three years postoperatively, the patient reported no pain, with no observed change in joint cavity space on radiography (Figure 4d).

Case 3

**Figure 5:**
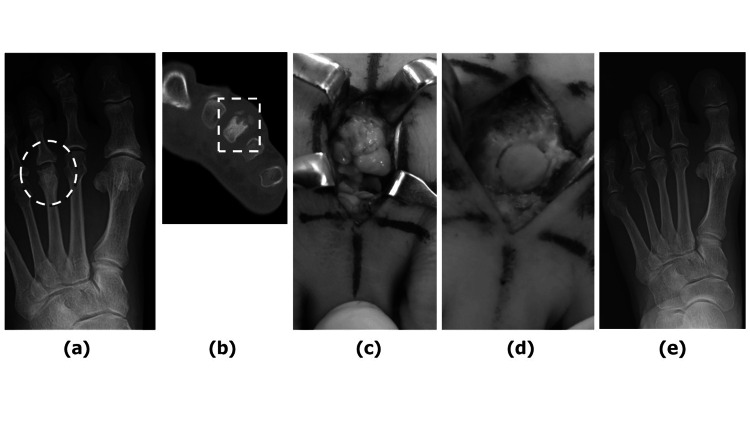
Case 3—Preoperative examination (a, b), intraoperative photographs (c, d), and postoperative radiograph (e) (a) AP view radiograph showing flattening of the third metatarsal head (indicated by a dotted circle) (b) Coronal plane CT scans showing the joint mouse on the dorsal side of the MTPJ (indicated by a dotted square) (c) Many osteophytes and joint mice are found around the metatarsal bone head (d) Outcome following OAT (e) AP view of the foot seven years postoperatively AP: Anteroposterior; MTPJ: Metatarsophalangeal joint; OAT: Osteochondral autograft transplantation

Our third case involved a 71-year-old woman who had a history of bruising on her left foot a year prior and was diagnosed with non-union of the left third metatarsal bone by her local doctor. On initial examination, the patient presented with local swelling and tenderness, and the ROM of the MTPJ of the third toe was limited to 20° of dorsiflexion and 10° of plantar flexion. Her VAS score was 6.0 points. Radiographs showed a crushed third metatarsal head, and CT scans revealed the development of osteophytes and a joint mouse (Figures 5a, 5b). The imaging findings corresponded to Smillie's stage 4 Freiberg disease. The patient exhibited normal bone mineral density in her lumbar spine and femur, measuring at 85% and 98%, respectively, according to the YAM value. The patient underwent OAT for collapse of the left third metatarsal head. During the procedure, many osteophytes and joint mice were found around the metatarsal bone head (Figure 5c). Once these were removed, the articular surface was examined and subchondral bone exposed. An osteochondral column (6.5 mm diameter, 10 mm in length) was harvested from the non-weight-bearing surface of the medial femoral condyle on the ipsilateral side and transplanted after a 9 mm hole was drilled into the articular surface of the metatarsal (Figure 5d). Histopathological diagnosis of the resected bone fragments did not reveal any findings suggestive of necrosis. Weight-bearing on the heel with a brace was permitted two weeks after surgery, progressing to full weight-bearing five weeks postoperatively. The JSSF scale improved from 67 points preoperatively to 95 points one year postoperatively, while the MTPJ ROM was 70° in dorsiflexion and 10° in plantar flexion. At seven years postoperatively, the patient remained pain-free, with no change in the joint cavity space observed on radiography (Figure 5e).

## Discussion

Freiberg disease, first described by Frieberg in 1914, manifests as avascular necrosis primarily affecting the metatarsal head, often seen in active adolescent female individuals [1,9]. While its etiology remains debated, it is commonly attributed to repeated mechanical stress leading to compromised blood flow and subsequent bone disintegration due to ischemia [4,10]. In middle-aged and elderly individuals, some presentations have been regarded as a separate disease. Young et al. reported a 55-year-old man with Freiberg infraction of the second metatarsal head [4]. They speculated that the shearing stress may be an important mechanism in the pathogenesis of adult Freiberg infraction because there was no necrotic bone but shearing type of separation in the pathological findings of metatarsal head. Tsujii et al. reported the case of a 77-year-old woman with clinical findings of Freiberg disease; however, pathological evaluation of the metatarsal head showed no necrotic tissue and was suggestive of a subchondral insufficiency fracture [5]. Furthermore, Torriani et al. investigated the MRI findings of subchondral fractures in metatarsal heads and reported the presence of two patterns that showed the early and late stages of the fractures [6]. They discussed subchondral insufficiency fractures and Freiberg disease as separate entities despite their similarities in clinical and imaging findings.

In our study, we presented three cases in middle-aged and elderly patients, one with hallux valgus and two with a history of trauma. Increased load on the second toe due to hallux valgus or trauma-induced stress may contribute to the onset of metatarsal head lesions. Histopathological evaluation revealed no necrotic findings, leading to a diagnosis of subchondral insufficiency fractures in these adult-onset cases. In general, subchondral insufficiency fractures tend to occur in the femoral head and talus. However, their occurrence may be associated with underlying bone fragility or predisposing systemic conditions [4]. In the present cases, there was no history of other fragility fractures, and one of the patients had bone density within the normal range, which may be atypical. Nelson et al. reported that 43% of patients with knee subchondral insufficiency fractures exhibit normal bone mineral density, suggesting that underlying osteoporosis may not always be present [11]. However, it is advisable to inquire about previous fractures and underlying diseases that may contribute to bone fragility during the diagnostic evaluation.

In this study, a subchondral insufficiency fracture was diagnosed, but due to similarities in clinical and imaging findings, the treatment approach aligned with that for Freiberg disease. In the early stages of Freiberg disease, conservative treatment mainly includes restricting movement and utilizing supportive braces [9,10]. However, surgical management is often recommended for advanced stage cases, instances of unsuccessful conservative treatment, and situations requiring early return to physical activity [12,13]. While drilling or debridement are options in the early stage, and resection or artificial joint replacement are options in the end stage, dorsal closed wedge metatarsal osteotomy (DCWMO) or OAT are mainly indicated in the advanced stage [10,14]. Incesoy et al. compared the outcomes of DCWMO and OAT for treating Freiberg disease [14]. They reported that the Sport score, activity score, and plantar flexion ROM were significantly greater in the OAT group, whereas the American Orthopedic Foot and Ankle Society (AOFAS) score was significantly greater in the DCWMO group, emphasizing the effectiveness of both treatments. DCWMO is widely used due to its potential for metatarsal bone head decompression and blood circulation improvement [13,15]. However, larger osteotomies may increase the risk of necrosis and non-union, with postoperative limitations the MTPJ ROM [16]. Additionally, it is not advisable for patients with hallux valgus, akin to one of our cases, as there is a risk of exacerbating hallux valgus due to metatarsal bone shortening as a result of the correction [5]. OAT may be a good option for such cases.

OAT as an operative treatment of Freiberg disease was first introduced by Hayashi et al., and has been noted for its efficacy, particularly in cases with extensive lesions, as it has a low risk of metatarsal shortening, osteonecrosis, and limited postoperative ROM, making it a viable option even for end stage Freiberg disease [16-18]. Hyakuda et al. reported three patients with stage 4 Freiberg disease treated with OAT of the metatarsal head [19]. While several reports have reported the usefulness of OAT in young patients with Freiberg disease, reports focusing on middle-aged and elderly patients remain scarce [10,12,20]. Kim et al. reported 12 cases with a mean age of 53.2 years who underwent OAT for Freiberg disease [16]. Despite including Smillie's stage 4 and 5, the outcomes were favorable. Moreover, postoperative clinical scores and ROM of the MTPJ were superior to those achieved with DCWMO. However, their diagnosis was limited to Freiberg disease without specifying pathological findings, such as necrosis. Thus, our study provides significant contributions by diagnosing a metatarsal head lesion as a subchondral insufficiency fracture occurring in middle age, offering detailed pathological findings, and reporting outcomes following OAT.

The biggest concern lies in the potential damage to healthy tissues during the cartilage column harvest for transplantation. While many reports advocate harvesting from the non-weight-bearing site of the femoral condyle , complications at the donor site, such as intra-articular hematoma, infection, and persistent pain, have been reported [10,12,20]. Tsuda et al. suggested a specific harvesting site anterior to the lateral sulcus on the outer edge of the pulley, which does not contact the lateral meniscus or tibial plateau [12]. However, they also pointed out that it is important to check for lesions in the knee joint preoperatively and to safely harvest the osteochondral column during surgery. In young patients, OAT has demonstrated early fusion of the articular surface and osteochondral column, facilitating a quicker return to physical activities [12]. However, concerns arise in middle-aged and elderly patients regarding the union rate of the osteochondral column and potential degeneration at the harvest site. Kim et al. stated that patients with systemic metabolic diseases, such as diabetes or chronic smokers, are not suitable for the procedure [16]. In our study, all three patients had no underlying disease or other risk factors. Although our patients experienced temporary discomfort in the knee joint postoperatively, it resolved over time without further complications, and they exhibited significant pain relief and improved clinical scores postoperatively. Radiographic findings also indicated favorable outcomes, suggesting the efficacy of OAT in middle-aged and elderly patients. Although a favorable outcome was obtained, the follow-up period was three to seven years. Therefore, further studies are needed to confirm long-term efficacy.

## Conclusions

We experienced three cases of subchondral insufficiency fractures of the metatarsal head in middle-aged to elderly women, presenting with symptoms similar to Freiberg disease. OAT led to significant pain relief and functional improvement in all cases. These findings suggest that OAT can be an effective treatment option for managing advanced subchondral insufficiency fractures of the metatarsal head, even in elderly patients.
